# The impact of aging and COVID-19 on our immune system: a high-resolution map from single cell analysis

**DOI:** 10.1007/s13238-020-00782-y

**Published:** 2020-09-07

**Authors:** Jing Yang, Hao Li

**Affiliations:** 1 School of Medicine, University of Electronic Science and Technology of China, 610054 Chengdu, China; 2 Department of Biochemistry and Biophysics, University of California, 94143 San Francisco, CA, USA

One key aspect of human aging is the dysregulation of the immune system. As we age, our body’s defense system weakens, leading to declined responses to infection by new pathogens and a decreased ability to become immunized (Goronzy and Weyand, 2013; Goronzy and Weyand, 2017). As a result, elderly people are more susceptible to the seasonal flu caused by the constantly mutating influenza viruses. Another manifestation of immune system aging is low grade chronic inflammation, which is linked to many age-related diseases (Glass et al., 2010; Ferrucci and Fabbri, 2018). Recent comprehensive characterization of cellular and molecular changes caused by aging in mammals further revealed the importance of immune system aging, as the up-regulation of genes involved in the inflammatory/immune response turned out to be a highly conserved signature across species and across different organs and tissues (Frenk and Houseley, 2018; Ma et al., 2020; Schaum et al., 2020; Tabula Muris, 2020). In addition, senolytic drugs targeting senescent cells which is a source of chronic inflammation showed great promise in retarding/preventing many age-related pathologies (Kirkland and Tchkonia, 2020).

With the raging COVID-19 pandemic ongoing, the interaction between aging and immune response to the pathogen SARS-CoV-2 has attracted great attention. It has been widely reported that elderly people are more susceptible to COVID-19 and, once infected, the consequence is much more severe. Among the infected, there is a higher rate of admission to ICU and higher rate of death for people aged 65 or older (Onder et al., 2020; Verity et al., 2020). The deaths associated with COVID-19 have been largely attributed to respiratory failure, sepsis, cardiac failure, kidney injury, or coagulopathy. Notably, substantial evidence suggests that in some patients the severity of the outcome is associated with dysregulated and excessive release of cytokines (Laing, 2020; Ye et al., 2020); such overreaction of the immune system can present a variety of symptoms ranging from fever and myalgias to severe cardiovascular, pulmonary, and renal damage.

So how does our immune system change due to aging? What drives the differential responses to infection between the young and old? What changes in the immune system in the elderly contribute to the increased susceptibility to and severity of COVID-19? In this issue, Zheng et al. set out to address these questions by systematically characterizing the changes in our immune system at the cellular and molecular level due to aging and due to COVID-19, either alone or in combination, using immune cells isolated from human peripheral blood and various single cell technologies. Their work presents a detailed view of how aging and COVID-19 affect our immune system and how the two interact with each other to amplify the effect, and suggests plausible mechanisms underlying the severity of COVID-19 in old patients.

Our immune system is complex. It consists of different types of cells residing in various tissues or circulating in the blood, with intricate regulatory mechanisms to control how they communicate and interact with each other. There are two basic types of immune responses in humans: the innate and the adaptive response. The innate immune response provides a first line of defense against the invader and is required for the activation of the adaptive response. Cells involved in the innate response include the natural killer (NK) cell, macrophage, neutrophil, monocyte (MC), dendritic cell (DC), etc., which can recognize and destroy the invading pathogen and infected cells nonspecifically. The adaptive immune response involves the T cell (TC) and B cell (BC) and mounts a response specific to the invading pathogen. Naive TCs and BCs express specific receptors (TCR and BCR) on their cell membrane that can recognize specific antigens. Upon binding to a specific antigen, immature B cells activate, proliferate, and differentiate into plasma cells to produce a specific antibody that can neutralize and guide the destruction of the pathogen. Naive T cells recognize the antigen presented by antigen presenting cells (such as DCs), proliferate, and differentiate into effector T cells, memory T cells, and regulatory T cells. Memory is formed so that the system can launch a swifter response upon future encounter of the same pathogen. At a finer resolution, the complexity is even more daunting as each cell type has multiple subpopulations that express specific sets of genes and are specialized to different tasks.

Given the complexity of the immune system, it is unsurprising that the aging phenotypes at the cellular/molecular level are also very complex. Instead of simple failure of particular components, aging brings about changes that cause dysregulation of the system (Nikolich-Zugich, 2018). Previous studies indicate that aging causes a decreased innate immune response by altering expression and/or function of innate immunity receptors and signal transduction, leading to defective activation and decreased chemotaxis, phagocytosis, and cytotoxicity (Solana et al., 2012). For the adaptive response, naive T cell decreases in blood and secondary lymphoid organs, accompanied by the increase of memory T cells (Nikolich-Zugich, 2018). This is intuitive; the naive lymphocytes differentiate into memory cells through interaction with the microbial universe throughout life, and the maintenance of the naive cells is impaired as we age. In addition, T cell and B cell receptor diversity was also found to decrease with age. Consistent with these findings, a recent systematic single cell study of aging in mice reveals that T cells decrease in the spleen, with a decreased naive T cell population and decreased TCR diversity (Tabula Muris 2020).

Although considerable knowledge has accumulated in the field, a comprehensive and high-resolution map of the human immune landscape in young versus old people is still lacking. There are numerous subpopulations of immune cells, each with their own specialized functions and complex interactions. A clear understanding of how the immune system responds to aging and COVID-19 requires a systematic characterization at the subpopulation level. How does the composition of the subpopulation change with aging and in response to COVID-19? How does the cellular state of each subpopulation change? What are the functional consequences of these changes?

To address the above questions, Zheng et al. collected the blood samples from young and old human subjects and isolated the peripheral blood mononuclear cells (PBMC), including BCs, TCs, NK cells, MCs, and DCs. Using a number of single cell technologies, they were able to quantify subpopulations in these different types of cells, characterize the changes in gene expression and chromosome accessibility in the subpopulations, and measure the clonal types and diversity of TCRs and BCRs. To characterize the interaction between aging and COVID-19, they also collected samples from young and old patients, either in the onset or recovered phase, and characterize the change of cell composition and gene expression.

To characterize the changes of cell populations and subpopulations, the authors used single cell RNA sequencing (scRNA-seq) and mass cytometry by time of flight (CyTOF). scRNA-seq measures genome-wide mRNA expression and the data can be used to identify different cell subpopulations, analyze differential gene expression, and infer transcriptional regulators. CyTOF is a variation of flow cytometry where antibodies are labeled by heavy isotopes and the abundance is measured through mass spectrometry. This method relies on antibody labeling of candidate proteins that are known to be present in different cell populations. The authors showed that the two independent methods both indicate that there is a decreased TC and BC populations and increased NK cells and MC populations (normalized by the total PBMC). On a finer resolution, the authors found a decrease in CD4^+^ and CD8^+^ naive T cell, as well as an increase of CD4^+^ effector memory T cells (Tem), CD8^+^ cytotoxic T cells (CTL), and exhausted T cells (Tex), in relative abundance in the respective subpopulation. The fraction of the classic CD14^+^ MC in PMBC clearly increased. Overall, the analyses of cell types and subpopulations indicate that aging shifts the immune cells towards more polarized and inflammatory populations. This general conclusion is consistent with previous findings in animal models and from human cytometry studies (Gounder et al., 2018; Nikolich-Zugich 2018). However, with higher resolution, the authors were able to quantify the changes of many subpopulations; the functional consequences of these changes on the immune regulatory networks are yet to be explored.

While the changes in the proportion of different cell types and their subpopulations undoubtedly contribute to the age-related decline of immune function, detailed characterization of the change of internal state of different subpopulations may provide mechanistic insight into the failure of different components and the mis-regulation of their interactions. To characterize the internal state of each cell subpopulation, the authors combined scRNA-seq and single cell ATAC-seq (the assay for transposase-accessible chromatin with sequencing) to map differential expression and differential chromosome accessibility in different cell subpopulations between the young and old subjects. They found differentially expressed genes (DEGs) common to all five immune cell types (TC, BC, DC, MC, and NK), as well as genes specific to a subset of these cell types. Zooming in with higher resolution, the authors found DEGs in different subpopulations of each of the cell types, e.g., DEGs in CD4^+^ T naive, Tcm, Tem, Treg, and Tex cells, and found genes common/specific to different subpopulations.

Besides the conserved gene expression signature in inflammatory pathways and oxidative stress response across different cell types, the authors found increased effector genes in TCs, and decreased genes involved in antigen presentation in DCs, suggestive of functional changes (such as the declined antigen presenting ability of DC) driven by intrinsic gene expression changes. Since the chromatin accessibility at gene promoters generally correlates with the expression level, their scATAC-seq data also allowed classification of different cell types and identification of differentially expressed transcription factors (DETs) based on the differential accessibility to the binding site (Schep et al., 2017), and the results largely corroborate the findings based on scRNA-seq. Among a number of DETs identified for different cell subpopulations, they found that some of the AP-1 family transcription factors (TFs), including JUNB and FOSL2, increased expression in all immune cells. JUNB is key transcriptional modulator of macrophage activation and serves as an activator for immune related genes (Fontana et al., 2015). FOSL2 was found to promote autoimmunity and inflammation by repressing regulatory T cells in mice (Renoux et al., 2020). Thus, the implication of these TFs is consistent with the DEG analysis. A number of bioinformatics tools have been developed to infer differentially expressed TFs from gene expression data (Aibar et al., 2017; Madsen et al., 2018; Roopra, 2020). It will be interesting to see if application of these tools to the scRNA-seq data will reveal similar TFs.

Previously, it was found that diversity of naive T cells shrinks considerably in the aged population, suggestive of declined adaptive immunity (Goronzy and Weyand, 2005). To examine the change of diversity of TCs and BCs, the authors analyzed B cell receptor (BCR) and T cell receptor (TCR) sequences in single cells. They quantified the distribution of clonal types and receptor diversity of different subpopulations in TCs and BCs. Consistent with previous findings, the authors found a clear decrease of diversity (measured by the number of distinguishing clones per 10^4^ cells), decreased number of unique clones, and increased clonal expansion (increased size of the maximum clone). The detailed sequence information also revealed that the use of the variable region genes is skewed. Going to finer resolution, the authors found decreased diversity in CD8^+^ Tem, possibly indicating decreased memory T cell survival due to aging. These age-associated changes of TCR and BCR may underlie the declined adaptive immune response in elderly people.

Having quantified the changes in PBMC due to aging, the authors went on to analyze the interaction between aging and COVID-19. Through a four-way comparison of young and old subjects with and without COVID-19, the authors found that COVID-19 infections lead to a depletion of TC and an increase of MC population, and in particular a depletion of CD4^+^ naive TC and increase of CD16^+^ MC, and that aged subjects with COVID-19 further exacerbated these trends. Since CD16^+^ MC has highly pro-inflammatory properties (e.g., high expression of proinflammatory cytokines) compared with the non-CD16 expressing classic monocytes and are expanded in various inflammatory diseases (Ziegler-Heitbrock, 2007), the observation of Zheng et al., suggests that aging and COVID-19 act synergistically to drain the adaptive immune system and to exacerbate the overly inflammatory response that may be partially responsible for the severity of the infection in aged patients. Further analysis of scRNA-seq data also suggests that aging leads to increased expression of the alternative cellular entry receptors for SARS-CoV-2, which may result in increased susceptibility to the infection. It will be very interesting to analyze whether these cellular entry receptors are under the control of the DETs, or are influenced by changed chromatin accessibility during aging. Future studies should examine the interactions between aging and other viral infections using a similar approach, to see whether the observed effect is general or specific to COVID-19.

To summarize, Zheng et al. systematically characterized the change of the human circulating immune cell landscape due to aging and COVID-19 and analyzed the interaction between the two. Using carefully designed experiments and various cutting-edge single cell technologies, they were able to quantify the changes in composition and the internal state of various immune cell types and their subpopulations. While many of their findings regarding the aging of the immune system are consistent with previous studies based on model organisms and flow cytometry studies of human immune cells, the authors presented a high resolution map with many new observations that potentially connect the changes at the cellular and molecular level to the macroscopic change of immune function. Their study also provided a vast amount of data that can be further explored by the research community to generate new testable hypotheses.

The big challenge going forward is to construct a causal and dynamic model of immune system aging, and to devise interventions based on mechanistic understanding. Charactering changes in various immune cell types and their subpopulations is an important first step towards that goal. Much more work is needed in the future to delineate changes that are drivers of the functional decline vs. those that are adaptive responses and even passive followers. Larger cohorts with a broader age distribution and longitudinal samples with finer temporal resolution should allow a better mapping of the dynamics of the change. In addition, characterization of PBMC should also couple with the characterization of lymphoid organs and other circulating factors in the blood, to better understand how the functional decline is shaped up by the altered interactions between these different components of the immune system. Recent studies of model organisms and other human samples also provide a great opportunity for comparative analysis across human tissues and across different mammalian species, to delineate conserved aging signatures and tissue/organ/species-specific features. Ultimately, mechanistic hypotheses connecting cellular and molecular changes to their functional consequences will need to be tested by targeted perturbation-response experiments.
